# Additive Manufacturing of Composite Polymers: Thermomechanical FEA and Experimental Study

**DOI:** 10.3390/ma17081912

**Published:** 2024-04-20

**Authors:** Saeed Behseresht, Young Ho Park

**Affiliations:** Department of Mechanical and Aerospace Engineering, New Mexico State University, Las Cruces, NM 88003, USA; behsaeed@nmsu.edu

**Keywords:** additive manufacturing, FEA, user subroutine, fused deposition modeling, composite polymers

## Abstract

This study presents a comprehensive approach for simulating the additive manufacturing process of semi-crystalline composite polymers using Fused Deposition Modeling (FDM). By combining thermomechanical Finite Element Analysis (FEA) with experimental validation, our main objective is to comprehend and model the complex behaviors of 50 wt.% carbon fiber-reinforced Polyphenylene Sulfide (CF PPS) during FDM printing. The simulations of the FDM process encompass various theoretical aspects, including heat transfer, orthotropic thermal properties, thermal dissipation mechanisms, polymer crystallization, anisotropic viscoelasticity, and material shrinkage. We utilize Abaqus user subroutines such as UMATHT for thermal orthotropic constitutive behavior, UEPACTIVATIONVOL for progressive activation of elements, and ORIENT for material orientation. Mechanical behavior is characterized using a Maxwell model for viscoelastic materials, incorporating a dual non-isothermal crystallization kinetics model within the UMAT subroutine. Our approach is validated by comparing nodal temperature distributions obtained from both the Abaqus built-in AM Modeler and our user subroutines, showing close agreement and demonstrating the effectiveness of our simulation methods. Experimental verification further confirms the accuracy of our simulation techniques. The mechanical analysis investigates residual stresses and distortions, with particular emphasis on the critical transverse in-plane stress component. This study offers valuable insights into accurately simulating thermomechanical behaviors in additive manufacturing of composite polymers.

## 1. Introduction

Additive manufacturing (AM) techniques have revolutionized the production of 3D parts with intricate geometries, concurrently streamlining product design costs in terms of both time and equipment [1,2,3,4,5,6]. Over the past decade, numerous additive manufacturing methods have emerged, including fused deposition modeling (FDM), powder bed fusion (PBF), and direct energy deposition (DED), among others. With the increased accessibility of cost-efficient printing machines and related equipment, AM has gained widespread use in rapid prototyping and is progressively being adopted in the production of parts across diverse industries such as construction, medical, automotive, and aerospace [7,8,9].

Of all the AM techniques, FDM utilizing semi-crystalline thermoplastic material in filament form as the raw material for producing 3D components has emerged as a preferred method for additive manufacturing due to its availability and affordability [10,11]. In the FDM process, a filament of thermoplastic material is fed into a hot extruder nozzle, where it is heated abovethe material’s glass transition temperature and close to its melting point. This technique contrasts with conventional methods within the same category, such as injection molding, which relies on pre-existing, costly molds and dies. During FDM, the molten filament is extruded through the heated nozzle and deposited directly onto a heated printing bed or onto a previously printed layer [12,13]. 

From a physical perspective, the FDM process is notably complex, involving a multitude of thermal phenomena. Heat transfer assumes a pivotal role throughout the process, particularly in determining the temperature distribution within the model, subsequently influencing the evolution of residual stresses and deformation, ultimately affecting the final integrity of the printed part [14,15,16,17]. Parts manufactured using FDM with semi-crystalline materials frequently exhibit residual stresses and shrinkage during the cooling phase following disposition [18]. The mechanical properties and integrity of a 3D-printed semi-crystalline polymer are significantly influenced by its thermal-mechanical response, cooling conditions, and the degree of crystallization [19,20]. Understanding these factors is critical for optimizing the fabrication process. The Finite Element Method (FEM) has proven to be a reliable and efficient approach for solving thermal and mechanical problems involved in FDM.

Over recent decades, various researchers have developed FEM-based techniques to simulate the FDM process with different thermoplastic materials [21], augmenting commonly used commercial software packages required for the FDM process by incorporating special-purpose user subroutines. Zhou et al. [22] developed a fully 3D thermal analysis for fused deposition modeling of polymers (ABS) using ANSYS APDL. In another study, they also devised an experimental method, employing infrared sensors, to measure the temperature of PLA polymer during deposition and simulated the process using ANSYS 17.2 to predict temperature and stress distribution [23]. Costa et al. [24] investigated heat transfer phenomena during FDM, including convection, radiation, and conduction, using Abaqus software 2023. Favaloro et al. [25] utilized newly added features in recent versions of Abaqus, including element activation and event series, among others, to simulate polymeric composite additive manufacturing. Zhang and Chou [26] examined the complex heat and mass transfer phenomena in FDM coupled with mechanical loading and phase changes, developing a finite element model using Ansys software. They then used this model to simulate temperature-induced residual stresses and part distortion. Yang and Zhang [27] established a numerical model of temperature and stress distribution for the forming process of FDM by finite element modeling method and birth-death element technique.

Utilizing these user subroutines in Abaqus usually demands proficiency in both thermomechanical behavior theory and Fortran programming. Particularly, studying the behavior of semi-crystalline polymers under various FDM conditions presents challenges in finite element analysis. This is due to the need to model temperature-dependent thermal expansion, thermo-mechanical properties of the polymer, and coupled crystallization kinetics, all evolving over time alongside finite element activation during printing. However, recent advancements have led to the partial integration of these user subroutine codes into an advanced additive manufacturing plug-in known as “Abaqus AM Modeler”, featuring a graphical user interface (GUI) [9]. The AM Modeler plug-in streamlines extensive coding by incorporating specialized functionalities tailored for additive manufacturing, such as element activation, and facilitates the easy handling of essential information, such as event series. 

In this study, we investigate the FDM process by incorporating various theoretical aspects, including heat transfer, polymer crystallization, anisotropic viscoelasticity, and anisotropic material shrinkage, with a specific emphasis on PPS material reinforced with 50 wt.% Carbon Fibers (CF). We performed a sequentially coupled thermal-mechanical analysis of the FDM process by integrating a comprehensive set of user subroutines into Abaqus (FEA-User Subroutines). We also conducted another sequentially coupled thermal-mechanical analysis of the FDM process by utilizing the Abaqus AM Modeler plug-in alongside selected user subroutines (FEA-AM Modeler). Our methodology undergoes rigorous validation against experimental data from leading research in the field, ensuring both efficiency and accuracy. Our findings also highlight the effectiveness and efficiency of integrating the AM Modeler with selected user subroutines, not only in terms of time and CPU utilization but also ease of assembly.

## 2. Materials and Methods 

### 2.1. Thermal Analysis

**Figure 1:**
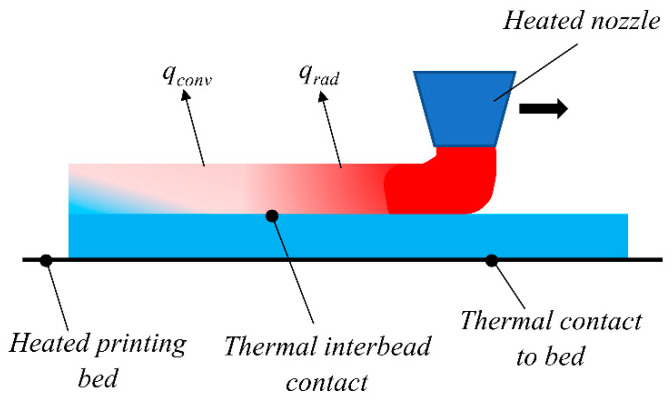
Schematic of the heat transfer phenomena during the FDM process.

The FDM process is based on the extrusion of heated thermoplastic polymers through a nozzle tip to continuously deposit layers onto a printing bed to fabricate parts layer by layer. Simultaneously, the previously deposited material begins to cool. Figure 1 provides a visual representation of the FDM method from the perspective of heat transfer. As the newly extruded material encounters the already deposited layers, a thermal connection forms between the roads (beads) of the polymer. The thermal resistance between the roads of the material hinges on the bonding efficiency, which evolves as the material cools to match the surrounding temperature [28].

Furthermore, thermal contact starts between the heated print platform and the initially printed layer. Thermal resistance plays a pivotal role in determining how effectively the printing bed can regulate thermal gradients across the first few layers of the printed component. On the external surfaces of the printed beads, heat dissipation due to convection and radiation comes into play, defining the cooling history of each added layer. These thermal dissipation mechanisms are profoundly influenced by the specific printer configuration employed and the geometry of the printed part.

The time spatial evolution of the temperature field can be presented by the heat equation [23]:

(1) $$\nabla \cdot \left(k\cdot \nabla T\right)+Q=\frac{\partial }{\partial t}\left(\rho \;C(T)\;T\right)$$

where *T* is temperature, *t* is time, *ρ* is the density, and *C* is the specific heat capacity. In modeling carbon fiber-reinforced anisotropic materials, orthotropic thermal properties are often assumed for simplification purposes. For an orthotropic material in a Cartesian coordinate system, Equation (1) becomes:

(2) $$\frac{\partial }{\partial x}\left(k_{x}(T)\frac{\partial T}{\partial x}\right)+\frac{\partial }{\partial y}\left(k_{y}(T)\frac{\partial T}{\partial y}\right)+\frac{\partial }{\partial z}\left(k_{z}(T)\frac{\partial T}{\partial z}\right)+Q=\frac{\partial }{\partial t}\left(\rho \;C(T)\;T\right)$$

where *x*, *y*, and *z* are the local spatial coordinates and *k_x_*, *k_y_*, and *k_z_* are the thermal conductivities in the different directions. To accurately describe heat dissipation to the surroundings, the radiation and convection have to be taken into account. The appropriate formulation for a boundary condition on an external surface is expressed as follows [29]:

(3) $$q=-k_{eff}\frac{\partial T}{\partial n}=h\left(T-T_{\infty }\right)+\varepsilon \sigma_{B}\left(T^{4}-T_{\infty }^{4}\right)$$

where *k_eff_* is the material conductivity in the direction of the surface normal *n*. In Equation (3), *h* is the film coefficient for convective heat transfer, *ε* the emissivity of the selected material, *σ_B_* the Stefan Bolzman coefficient, and $T_{\infty }$ the temperature of boundaries of the surface. The contact between printed beads of material can be formulated as follows [30]:

(4) $$q=-k_{eff,1}\frac{\partial T_{1}}{\partial n}=h_{c}\left(T_{1}-T_{2}\right)=-k_{eff,2}\frac{\partial T_{2}}{\partial n}$$

where *h_c_* is the contact conductance of the interface and *k_eff_*_,1_ and *k_eff_*_,2_ are the effective conductivities perpendicular to the surfaces 1 and 2 that meet one another. Considering the temperatures of the in-contact surfaces *T*_1_ and *T*_2_, it implements the heat transfer throughout the interface. In the case of a perfect contact (*h_c_*→∞), the surface temperatures of the contacting objects become equal, yielding *T*_1_ = *T*_2_.

It is worth noting that the internal heat source *Q*, which accounts for the exothermic crystallization process, can be ignored due to its insignificance compared to the heat dissipated to the surrounding environment. The thermal conductivities of 50 wt.% CF PPS material across temperature ranges from room temperature to material extrusion temperature of 300 °C can be described as functions of temperature (*T*) in three principal directions [31]:

(5) $$\begin{array}{l} k_{x}(T)=0.0041\;T+0.7018\\ k_{y}(T)=0.0011\;T+0.3913\\ k_{z}=0.3768 \end{array}$$

Additionally, the coefficient of thermal expansion (CTE) is also dependent on temperature due to the phase change from a molten, amorphous to a fully crystallized and solid state. This dependency on temperature can be represented as follows [31]:

(6) C(T)=2.8543 T+59.228

### 2.2. Polymer Crystallization Kinetics

Since PPS is a semi-crystalline polymer, it is essential to model its crystallization behavior because the crystallization process changes the material’s mechanical properties and introduces additional shrinkage. To capture the crystallization behavior of PPS, we employed the dual non-isothermal, Avrami-type crystallization kinetics model [32]:

(7) $$X(t,T,p)=X_{\infty }[w_{1}F(t,T)+w_{2}F(t,T)]$$

where

(8) $$F_{i}=1-\exp\left[-C_{i1}\int_{0}^{t}T\cdot \exp\left\{-\left[\frac{C_{i2}}{T-T_{g}+T_{add,i}}+\frac{C_{i3}}{T{\left(T_{m.i}-T\right)}^{2}}\right]\right\}\cdot n_{i}\tau^{n_{i}-1}d\tau \right],\;i=1,2$$

The degree of crystallinity *X* is determined by a weighted combination of two independent crystallization processes, *F*_1_ and *F*_2_ (*w*_1_ + *w*_2_ = 1), bounded by a maximum crystallinity *X*_∞_.

### 2.3. Viscoelastic Model of Orthotropic Materials

Unlike linear elastic materials, the behavior of viscoelastic materials depends on their loading history. A common experimental technique to assess the viscoelastic properties involves conducting a relaxation test. This characterization may be conducted by a uniaxial tensile test with a constant strain *ε*_0_ while measuring the stress *σ*(*t*) as a function of time.

(9) $$\sigma (t)=\int_{0}^{t}E(t-\tau )\frac{d\varepsilon }{d\tau }d\tau$$

Expanding Equation (9) to three dimensions involves substituting the 1D relaxation modulus with a relaxation stiffness matrix, employing Voigt Notation. The total stress *σ* at time *t* can be expressed using the convolution integral:

(10) $$\sigma_{i}(t)=\int_{0}^{t}C_{ij}(t-\tau )\frac{d\varepsilon_{j}}{d\tau }d\tau$$

where *C_ij_* are the components of the time-dependent (relaxation) stiffness matrix. The components of the stiffness matrix, *C_ij_,* are assumed to be expressed by a Prony series as follows:

(11) $$C_{ij}=C_{ij0}+\sum_{w=1}^{N}C_{ijw}e^{\left(-\frac{t}{\lambda_{ijw}}\right)}$$

where *C_ijw_* represents the unrelaxed parts of the stiffness matrix components, *C_ij_*_0_ describes the relaxed contributions, and *N* denotes the number of Maxwell elements represented by the Prony series. In Equation (11), the relaxation time *λ_ijw_* associated with each of the *N* Maxwell elements governs the overall relaxation behavior. The stress at any given time *t* can be obtained by inserting Equation (11) into Equation (10), which can be rearranged as

(12) $$\sigma_{i}(t)=\int_{0}^{t}\left(C_{ij0}+\sum_{w=1}^{N}C_{ijw}e^{\left(-\frac{t-\tau }{\lambda_{ijw}}\right)}\right)\frac{d\varepsilon_{j}}{d\tau }d\tau$$

### 2.4. Determination of the Model Parameter

With creep experiments conducted in all directions, determining the parameters of the material model is straightforward. The stress-strain relationship is defined as:

(13) $$\left(\begin{array}{c} \sigma_{11}\\ \sigma_{22}\\ \sigma_{33}\\ \sigma_{12}\\ \sigma_{13}\\ \sigma_{23} \end{array}\right)=\left[\begin{array}{cccccc} C_{11} & C_{12} & C_{13} & 0 & 0 & 0\\ C_{12} & C_{22} & C_{23} & 0 & 0 & 0\\ C_{13} & C_{23} & C_{33} & 0 & 0 & 0\\ 0 & 0 & 0 & C_{44} & 0 & 0\\ 0 & 0 & 0 & 0 & C_{55} & 0\\ 0 & 0 & 0 & 0 & 0 & C_{66} \end{array}\right]\left(\begin{array}{c} \varepsilon_{11}\\ \varepsilon_{22}\\ \varepsilon_{33}\\ \varepsilon_{12}\\ \varepsilon_{13}\\ \varepsilon_{23} \end{array}\right)$$

where:

(14) $$C_{11}=\frac{E_{1}(1-v_{23}v_{32})}{\Delta },\;C_{22}=\frac{E_{2}(1-v_{13}v_{31})}{\Delta },\;C_{33}=\frac{E_{3}(1-v_{12}v_{21})}{\Delta }$$

(15) $$C_{12}=\frac{E_{1}(v_{21}+v_{31}v_{23})}{\Delta },\;C_{13}=\frac{E_{1}(v_{31}+v_{21}v_{32})}{\Delta },\;C_{23}=\frac{E_{2}(v_{32}+v_{12}v_{31})}{\Delta }$$

(16) $$C_{44}=G_{12},\;C_{55}=G_{13},\;C_{66}=G_{23}$$

(17) $$\Delta =1-v_{12}v_{21}-v_{23}v_{32}-v_{31}v_{13}-2v_{21}v_{32}v_{13}$$

The orthotropic stiffness matrix in Equation (13) consists of nine components that are dependent on Young’s moduli, Poisson’s ratios, and shear moduli of the material as defined in Equations (14)–(17). These properties are time and temperature-dependent and represented by a fitted Prony series model obtained from the experimental master curves [31]. Reference [31] provides a comprehensive experimental procedure for extracting mechanical properties such as Young’s moduli, Poisson’s ratios, and shear moduli. Additionally, detailed methodologies for conducting experiments, including digital image correlation (DIC) experiments for material shrinkage, sagging experiments for gravity effects, and stress relaxation time-temperature superposition (TTS) experiments for relaxation moduli, are thoroughly described in Reference [31]. 

### 2.5. Anisotropic Shrinkage

For semi-crystalline materials like PPS, it is essential to model both the anisotropic thermomechanical and crystallization shrinkage. In fiber-reinforced materials, the orientation of the fibers impacts shrinkage differently in different directions. When fibers are aligned with the printing direction (1-direction), they either inhibit or reduce crystallization-induced shrinkage, while a large amount of the material shrinkage occurs in the transverse direction [33]. In this study, we assume that material shrinkage due to crystallization occurs solely in the transverse direction. As a result, the total inelastic incremental strain in the stress-free state is a combination of thermal and crystallization strain, as shown below:

(18) $$\Delta \varepsilon_{1}^{inel}=\Delta \varepsilon^{th}=\alpha_{1}\Delta T$$

$$\Delta \varepsilon_{i}^{inel}=\alpha_{i}\Delta T+\varepsilon_{i}^{cr}\frac{X_{Diff}}{X_{\infty }},\;i=2,3$$

where *α_i_* the coefficient of thermal expansion and $\varepsilon_{i}^{cr}$ is the strain due to crystallization, and *X_Diff_/X*_∞_ is the relative incremental change in crystallinity. 

## 3. Finite Element Simulation Technique

Finite element analysis has always been an efficient way to solve different analyses, from thermal to mechanical, and in a wide variety of fields using several commercially available software such as Ansys, Abaqus, etc. [34,35]. Following the thermal modeling of the FDM process in the previous section, we now discuss the details of finite element simulation techniques employed to capture the physical phenomena during the FDM process. In this section, we provide an overview of the main approaches employed for the implementation of Abaqus user subroutines (FEA-User Subroutines) and AM Modelers (FEA-AM Modelers).

### 3.1. FEA-User Subroutines

#### 3.1.1. UMATHT

In order to implement the thermal orthotropic constitutive material behavior of 50 wt.% CF PPS, as previously outlined in Section 2, we have developed and utilized the UMATHT user subroutine within Abaqus. This process involves defining thermal conductivities and the Coefficient of Thermal Expansion (CTE) as functions of temperature (as in Equations (1) and (2), respectively). Then incremental changes in internal energy (*dU*) are determined, and the subsequent total internal energy value is updated:

(19) $$\begin{array}{l} dU=C(T)\;dT\\ U=U+dU \end{array}$$

Next, vector fluxes in three different material directions are computed using Fourier’s law as follows:

(20) $$f_{x}=-k_{x}\frac{\partial T}{\partial x},\;f_{y}=-k_{y}\frac{\partial T}{\partial y},\;f_{z}=-k_{z}\frac{\partial T}{\partial z}$$

Finally, we determine the derivative of the heat flux with respect to spatial gradients of temperature and the variation of heat flux with respect to temperature, which is essential for the UMATHT subroutine:

(21) $$\frac{\partial f_{x}}{\partial \left(\frac{\partial T}{\partial x}\right)}=-k_{x};\frac{\partial f_{y}}{\partial \left(\frac{\partial T}{\partial y}\right)}=-k_{y};\frac{\partial f_{z}}{\partial \left(\frac{\partial T}{\partial z}\right)}=-k_{z}$$

(22) $$\frac{\partial f_{x}}{\partial T}=-\alpha \frac{\partial T}{\partial x};\frac{\partial f_{y}}{\partial T}=-\beta \frac{\partial T}{\partial y};\frac{\partial f_{z}}{\partial T}=-\gamma \frac{\partial T}{\partial z}=0$$

where *α*, *β*, and *γ* represent the slopes of thermal conductivities as linear functions of temperature in the *x*, *y*, and *z* directions, respectively.

#### 3.1.2. UEPACTIVATIONVOL

An intrinsic aspect of the FDM process involves the progressive addition of material, starting from the nozzle to the printing bed during the initial layer deposition and subsequently onto the previously deposited material in subsequent layers. Abaqus versions since 2017 have introduced the UEPACTIVATIONVOL user subroutine, a potent tool that enables the progressive activation of elements during analysis as a function of time and position. This user subroutine serves as a useful tool for simulating the FDM AM process.

To provide UEPACTIVATIONVOL with the necessary data, the physical machine’s tool path is provided as an event series, typically extracted from slicer software such as Creality v4.3.8 used in this study. This event series encompasses critical information, including the extruder head’s position at any given time, print speed, and the direction of nozzle movement. Essentially, it is the same as the G-Code generated by the slicer software. In the FDM process, material activation occurs in the form of beads, each comprising multiple elements based on the mesh size. These material beads are activated sequentially, and all the elements of a bead are activated at a specific time, referred to as the activation time. The activation time for each bead, which aligns with the elements within that bead, is passed to the UEPACTIVATIONVOL subroutine using common blocks from the ORIENT user subroutine, detailed in the subsequent section.

#### 3.1.3. ORIENT

Given that 50 wt.% CF PPS is an orthotropic material with different thermal properties in three mutually perpendicular directions; we have developed the ORIENT user subroutine to define material orientation at specific points within the model. This subroutine is called at the onset of the analysis and once for each material point. It incorporates an algorithm for reading event series data from a text file provided by the physical machine in G-Code format. The subroutine also assigns activation times and material orientation, defined using the start and end locations of material beads, to individual elements. This activation time is then provided to the UEPACTIVATIONVOL user subroutine that will be used for progressive element activation. To optimize computational efficiency, the algorithm for reading event series data and assigning it to elements is executed only for one integration point of each single element. The extracted information is then stored in global variables within the UEXTERNALDB user subroutine for use by other integration points of the same element, reducing computation time.

#### 3.1.4. UEXTERNALDB

UEXTERNALDB is typically utilized for reading data from files, storing data in external files, and facilitating communication between user subroutines. In this context, a UEXTERNALDB user subroutine has been implemented to store activation times and material orientation for each element and subsequently share this vital information with other user subroutines, such as UEPACTIVATIONVOL, using common blocks.

#### 3.1.5. UMAT

To model the mechanical behavior and governing constitutive equations of 50 wt.%CF PPS, a maxwell model for viscoelastic materials is implemented into a UMAT user subroutine. As PPS is a semi-crystalline polymeric material, the crystallization kinetics behavior is crucial because the crystallization process affects the mechanical properties of the material and causes further shrinkage. The crystallization behavior of the CF/PPS material is implemented in the UMAT user subroutine by a non-isothermal crystallization kinetics model described in Equations (7) and (8) in Section 2.2. In Equation (8), a total number of 11 fitting parameters was determined based on the experimental data *p* = (*w*_1_, *C_i_*_1_, *C_i_*_2_, *C_i_*_3_, *C_i_*_1_, *T_add_*_,*i*_, and *T_m_*_,*i*_), *i* = 1, 2 as described in Reference [32].

During the FDM process, the material immediately starts to cool down and consequently shrink. Since the material being deposited is constrained by the print bed and previously deposited layers, the material is unable to shrink freely, and stresses start to build up inside the printed part. However, since the material is considered thermoviscoelastic, stress relaxation occurs as well. To accurately model these stresses and the corresponding warpages, a unique approach is required to account simultaneously for both the stress accumulation and the stress relaxation. Besides, such a model must be able to describe the anisotropy of material, and the cooling rate-dependent imposed shrinkage strain histories must be captured accurately. In the present implementation, a numerical recursive scheme [36,37] is adopted that meets these requirements.

(23) $$\sigma_{i}(t_{n})=\sigma_{i0}(t_{n})+\sum_{j=1}^{6}\sum_{w=1}^{N}s_{ijw}(t_{n}),\;i,j=1,2,\ldots ,6$$

where

(24) $$\sigma_{i0}(t_{n})=\sigma_{i0}(t_{n-1})+f(X(t_{n}))C_{ij0}\Delta \varepsilon_{j,n}^{eff}$$

(25) $$s_{jwi}(t_{n})=\exp\left(-\frac{\xi_{ij}(t_{n})-\xi_{ij}(t_{n-1})}{\lambda_{ijw}}\right)s_{ijw}(t_{n-1})+f(X(t_{n}))C_{ijw}h_{ijw}(\Delta t_{n})\Delta \varepsilon_{j,n}^{eff}$$

This approach is based on the assumption that the stiffness matrix components, *C_ij_*, can be characterized using the Prony Series type model. This model incorporates one relaxed part, *C_ij0_*, and *N* number of unrelaxed stiffness matrix components, *C_ijw_*, where *w* ranges from 1 to *N*, with *N* being the number of employed Maxwell elements. Consequently, six components of stress are decomposed into both single relaxed parts and several unrelaxed parts. For all Six components of stress, *σ*_*i*_ in Equation (23), the relaxed part *σ*_*i*0_ contains all relaxed stresses from previous increments *σ*_*i*0_(*t*_*n*−1_) and the contribution of the current increment, defined by the crystallization-dependent factor, *f*(*X*(*t_n_*)) along with the incremental effective strains, $\Delta \varepsilon_{j,n}^{eff}$. The unrelaxed stress components, *s_ijw_*, also contain two parts, with the first part accounting for the stress relaxation of previously accumulated stresses based on the relaxation times, *λ_ijw_*, and the current reduced times, *ξ_ij_*. This process reflects time-temperature superposition and captures the material’s relaxation behavior at the current temperature. The second part describes how unrelaxed stresses accumulate based on the material shrinkage behavior, defined again by the crystallization-dependent pre-factor, *f*(*X*(*t_n_*)), and the incremental effective strains, $\Delta \varepsilon_{j,n}^{eff}$. The characteristic function, ℎ*_ijw_*(Δ*t_n_*), plays a crucial role in accounting for the stress relaxation occurring within the increment. With its two parts, *s_ijw_* satisfies the need to simultaneously describe stress relaxation and stress generation, as discussed above. Additional information on this recursive numerical approach and its derivation can be found in in references [36,37,38]. 

### 3.2. FEA-AM Modeler

In more recent versions of Abaqus, starting from 2018 [9], the AM Modeler plug-in has introduced new features for simulating various additive manufacturing processes. The method proposed in this study, combining the FEA-AM Modeler approach, employs both the AM Modeler plug-in and selected user subroutines to facilitate and expedite the simulation of the FDM process, thereby reducing the complexity and computational cost of analysis. In the FDM process, the material exits the nozzle in a molten state and rapidly cools to room temperature within seconds, resulting in significant temperature gradients that lead to unexpected residual stresses and geometric distortions. Consequently, a full thermo-mechanical analysis was conducted. 

**Figure 2:**
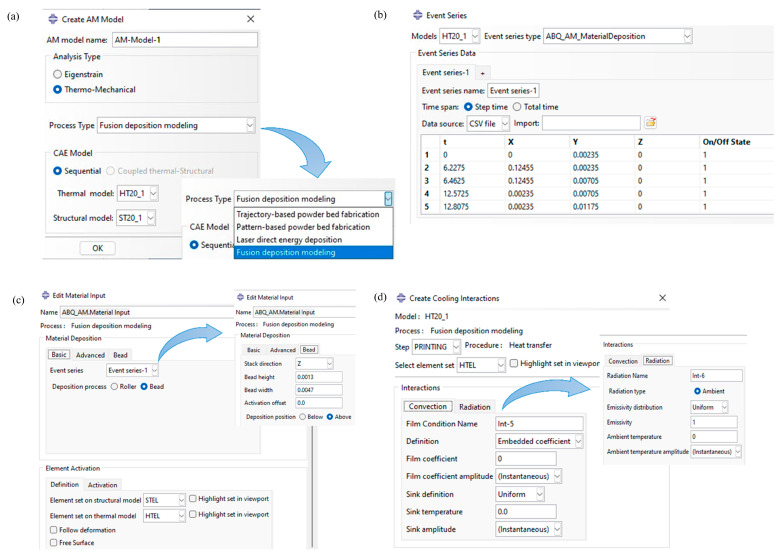
Application of AM Modeler in FDM: (**a**) Type of AM process, (**b**) Event series input, (**c**) Element activation profile, (**d**) Thermal boundary conditions.

After selecting the type of AM process (FDM) as shown in Figure 2a, to replicate the controlled deposition of raw materials, we employed the event series functionality that facilitates the progressive activation of elements. The tool path of the physical printer, which is provided directly from the g-code file, is then fed into the AM modeler to simulate the element activation process. A snapped piece of the used event series of the test plate is depicted in Figure 2b. The deposition process takes the form of rollers or beads, depending on the analysis type. In the case of FDM, the material is deposited in rectangular beads for simplicity. In this study, bead width and height are defined based on the physical printer specifications. For our current model, we considered a bead width of 4.7 mm and a bead height matching the layer thickness of 1.3 mm, as shown in Figure 2c. The element set is defined as a simple cubic voxel mesh, and the primary geometry is activated based on event series data. 

To apply convection and radiation heat transfer boundary conditions in the FDM process, we used the cooling function to cooling functionality, as depicted in Figure 2d. In FDM, material elements are gradually activated, leading to the progressive activation of new faces and the overlaying of previously activated element faces with newly activated ones. Boundary conditions are exclusively applied to active faces and are automatically removed when faces become covered by other elements. 

**Figure 3:**
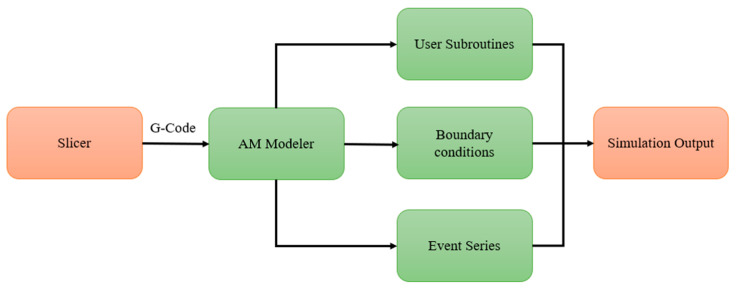
Schematic of the proposed model for FDM process simulation in Abaqus.

The integration of selected user subroutines and AM Modeler has been seamlessly implemented to facilitate robust communication and, consequently, the accurate simulation of thermo-mechanical analysis throughout the FDM process. Figure 3 provides a visual representation of the interconnected structure of user subroutines with AM Modeler. 

The g-code file generated by the slicer software is inputted into the AM Modeler. The AM Modeler then supplies selected user subroutines, such as ORIENT, that require tool path information for material orientation. It also executes material activation based on event series information (Figure 2c) and applies boundary conditions, as shown in Figure 2d. 

## 4. Verification and Validation of FE Simulation Techniques

In this section, we conduct a comparative examination of nodal temperature distributions obtained from both FEA-AM Modeler and FEA-User Subroutines for thermal analysis, as well as stress and displacement results for structural analysis. First, the results obtained from conventional user subroutines are validated using experimental data obtained from conducted experiments (for thermal analysis) and existing data in the literature (for structural analysis). Then, the proposed FEA-AM Modeler is assessed by comparing the results of thermal and structural analyses obtained from this approach with those obtained from the proposed FEA-User Subroutines. 

### 4.1. Thermal Analysis

To validate the accuracy of the proposed Finite Element (FE) simulation techniques, we examined the FDM process for 50 wt.% CF PPS using both Abaqus user subroutines and the FEA-AM Modeler. Our FE model was constructed using 3D solid elements with the DC3D8 type, specifically for sequentially coupled heat transfer analysis.

#### 4.1.1. FE Simulation Model

**Figure 4:**
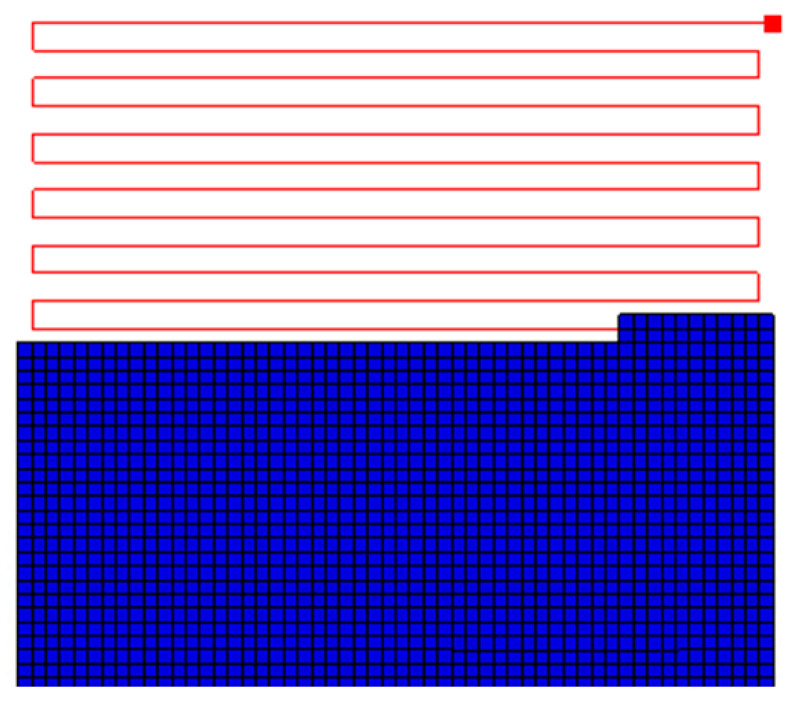
Meshed plate and toolpath of the physical machine.

Figure 4 shows the FE model, complete with mesh and tool path for the initial layer (0°), highlighted by the red lines. The next layer is deposited atop the first layer with a 90-degree orientation (90°). The FE model is a plate measuring 125 mm × 125 mm × 2.6 mm. Each material bead consists of two elements in width and two elements in height, resulting in a total of 11,664 elements. The print speed for PPS material is set at 20 mm/s. The analysis comprises three steps: the printing process, during which material is activated at the melt temperature and subsequently cools to ambient temperature; the cooling step on the printing bed, where the printed plate remains on the hot printed bed for 5 min; and the removal of the printed plate from the hotbed, allowing it to cool to room temperature over 10 min.

**Figure 5:**
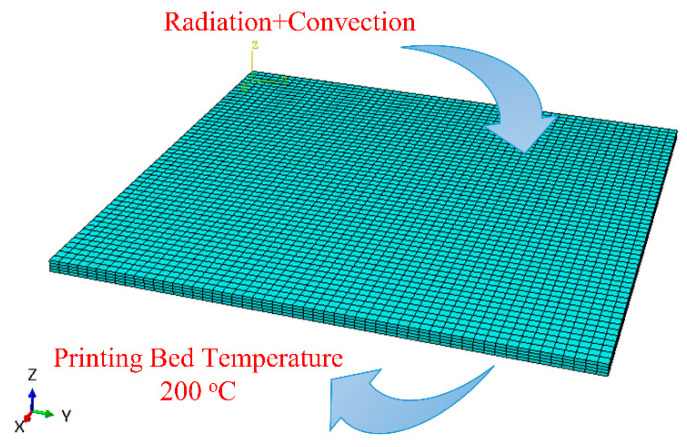
Thermal boundary conditions.

Boundary conditions include a hot printing bed temperature of 200 °C applied to the bottom face of the plate, coupled with convection and radiation boundary conditions to account for heat transfer to the surrounding environment, as shown in Figure 5. The surrounding temperature is 25 °C, and the material’s emissivity for radiation is 0.97. A comprehensive summary of all simulation parameters is available in Table 1.

#### 4.1.2. Experimental Verification

**Figure 6:**
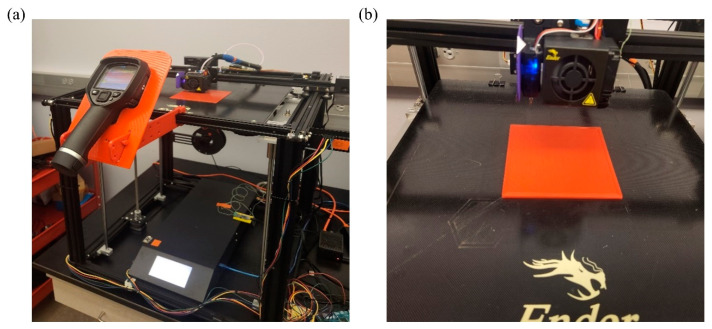
(**a**) The thermal camera mounted on the printer; (**b**) 3D printed plate with PLA.

In this section, we validate the FDM process simulation using user subroutines (FEA-User Subroutines) through a comparative experimental analysis of nodal temperature distribution in a 3D printed component. The experimental setup for the FDM printing process can be seen in Figure 6a. We utilize a 3D-printed plate with a geometry similar to that described in Section 4.1.1 but fabricated from a different material. The plate, shown in Figure 6b, measures 120 mm × 120 mm × 5 mm and is made of Polylactic Acid (PLA). PLA was selected for this study due to its widespread availability and sustainability for 3D printing [39]. Printing parameters are listed in Table 2.

**Figure 7:**
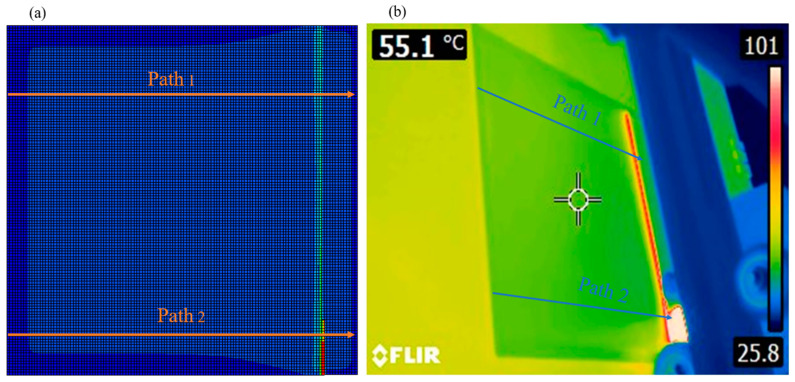
(**a**) Selected nodal paths for thermal analysis verification; (**b**) A thermal image captured during the FDM printing process.

The nodal temperature distribution was measured along two specific paths on Layer 4 of the 3D-printed plate. The selected nodal paths for temperature distribution on the 4th layer of the plate are shown in Figure 7a.

The simulated nodal temperature distributions were obtained from Finite Element Analysis conducted using the conventional FEA-User Subroutines. Temperature measurements on Layer 4 of the plate were carried out using the FLIR E8-XT thermal infrared camera. The FLIR E8-XT infrared camera captured images (see Figure 7b), which were then recorded in a digital system operating at a frequency of 9 Hz, ensuring reliable data. 

**Figure 8:**
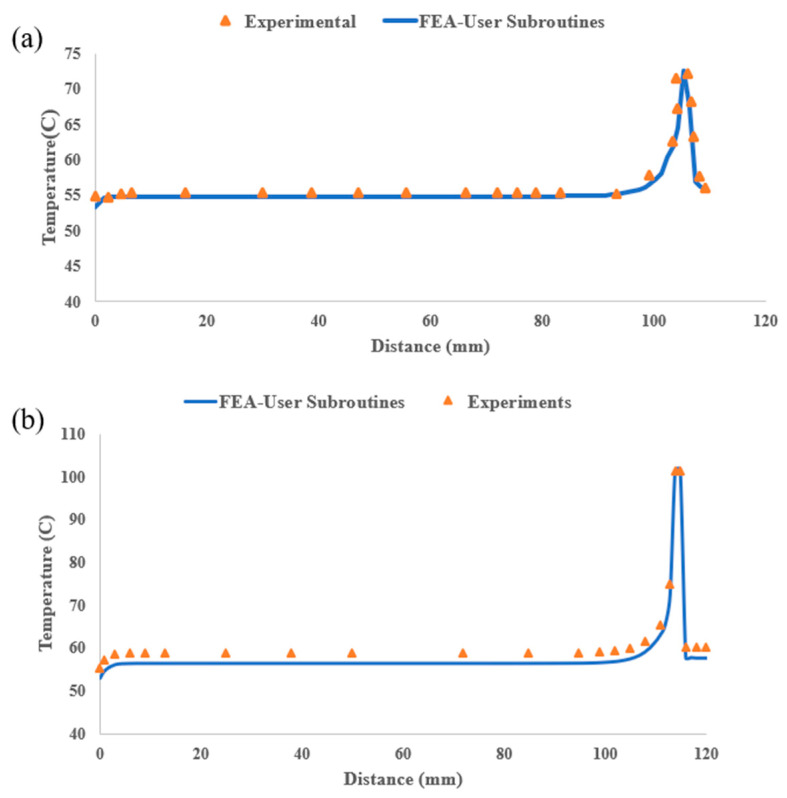
Nodal temperature distribution along selected paths. (**a**) Path 1; (**b**) Path 2.

The FLIR E8-XT camera maintains a thermal precision of ±2 °C or ±2%, covering a temperature range of −20 to 550 °C, with an adjustable emissivity set to 0.97 to be consistent with simulation parameters outlined in Table 2. Positioned at a distance of 20 cm from the printed part, the camera captures images at a resolution of 320 × 240 pixels. The results are presented in Figure 8, where a notable agreement is observed between the results obtained through both methodologies along path 1, shown in Figure 8a, and path 2, depicted in Figure 8b. Along both paths, the layer-by-layer printing process initiates from the origin and progresses in the y-direction from left to right. This progression creates a temperature gradient among the nodal points. On the left-hand side, temperatures are lower, gradually increasing as we move towards the right, where new material has just been deposited. The lower temperature near the far-left edge is a result of heat dissipation from both the top and left surfaces of the plate in that area. Conversely, for the central nodes located further from the left edge, heat dissipation primarily occurs from the top surface, resulting in slightly higher temperatures at these nodes. These temperatures remain relatively constant as we move further away from the newly deposited material on the right-hand side, where the temperature is at its maximum due to the recent addition of material that has not yet cooled down. Beyond this point, the temperature sharply decreases to reach the equilibrium temperature of the previous layer, which is not yet covered by the new layer. The maximum temperature along both paths occurs at the location of the recently deposited road of PLA. However, since the current road of polymer is deposited from left to right, the maximum temperature of path 2 (~102 °C) is higher than that of path 1 (~73 °C) as the material is recently deposited at this location.

Figure 8 demonstrates a clear correspondence between the experimental results and the results obtained from traditional FEA-User Subroutines. Therefore, FEA-User Subroutines can be effectively employed to evaluate the proposed FEA-AM Modeler methodology.

#### 4.1.3. Verification of FEA-AM Modeler

**Figure 9:**
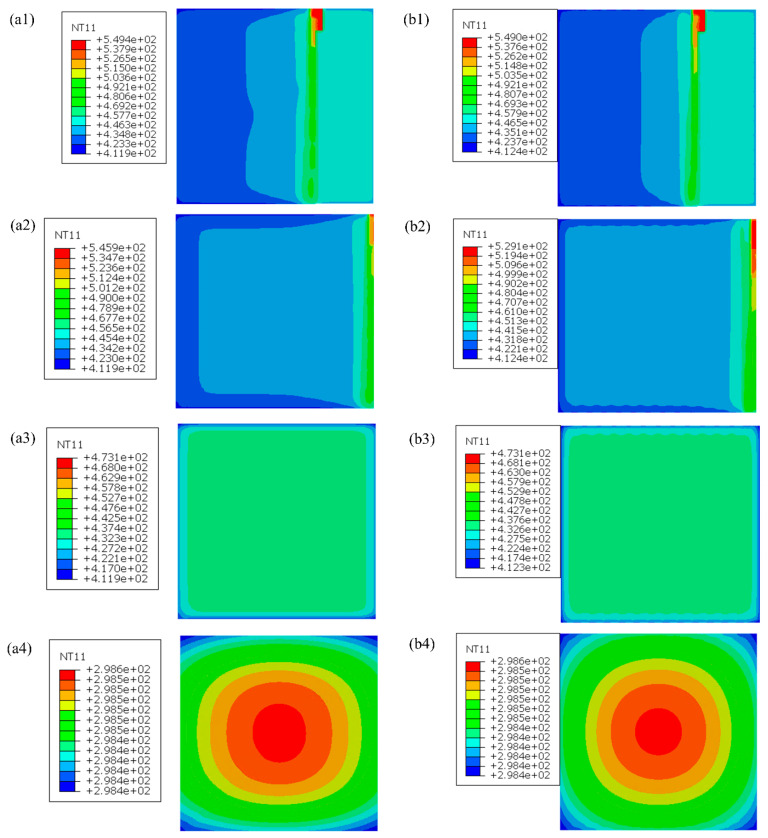
Nodal temperature distribution in different steps of analysis for FEA-User Subroutines (**a**) and FEA-AM Modeler (**b**): (**1**) Printing step, (**2**) end of printing step, (**3**) end of cooling step on bed, and (**4**) end of cooling step off printing bed.

The nodal temperature distributions, representing various stages of the analysis, were obtained from both the FEA-AM Modeler (right column) and Abaqus user subroutines (left column) and are visually presented in Figure 9. The first row shows the temperature distribution during the printing step, while the second row represents the temperature profile of the plate at the end of the printing step. The third row depicts the temperature distribution at the end of the cooling step on the printing bed, and the fourth row displays the temperature distribution at the end of the cooling step off the printing bed. From the contour plots, it is evident that the results of thermal analysis obtained from both FEA- User Subroutines and the FEA-AM Modeler exhibit excellent agreement with a maximum error of less than 3%, which is quite acceptable in engineering applications.

**Figure 10:**
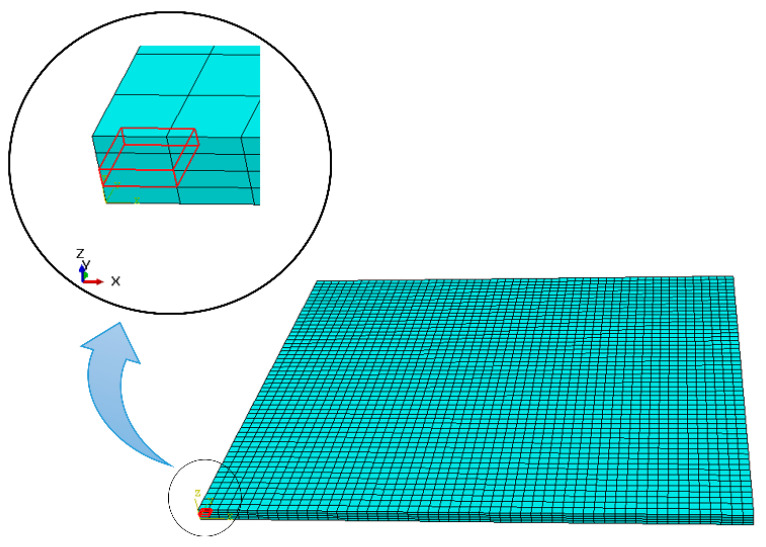
Candidate element for thermal analysis.

**Figure 11:**
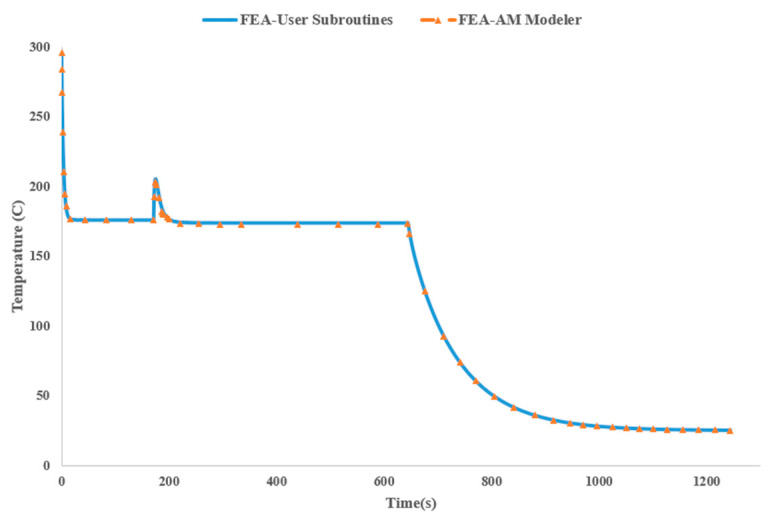
Nodal temperature distribution of element of interest with respect to time.

To further verify the consistency of results between the FEA-AM Modeler and the traditional FEA-User Subroutines approach, we charted the temperature changes over time for an element (as depicted in Figure 10) activated at the outset of the analysis (Figure 11). This specific element resides within the first bead, which is activated precisely at the beginning of the analysis at 300 °C, coinciding with the extrusion temperature of PPS. Due to the substantial temperature difference between the extrusion temperature and the low ambient temperature, coupled with a high film coefficient of 30 W/mK for convection boundary conditions, the temperature of the element sharply drops to approximately 180 °C and remains constant until the next layer is deposited at *t* = 172 s. At this point, the temperature begins to rise as newly added material causes the already deposited material to melt. Then, the temperature gradually decreases, remaining constant until the cooling step is completed on the bed. This steady-state temperature distribution is attributed to the equilibrium between the heat flux generated by the elevated printing bed temperature and the heat dissipated from the top surface into the surrounding environment. Finally, during the last step of the analysis, the temperature in the element gradually decreases as the printing bed temperature is eliminated, allowing the entire part to cool down to ambient temperature.

**Figure 12:**
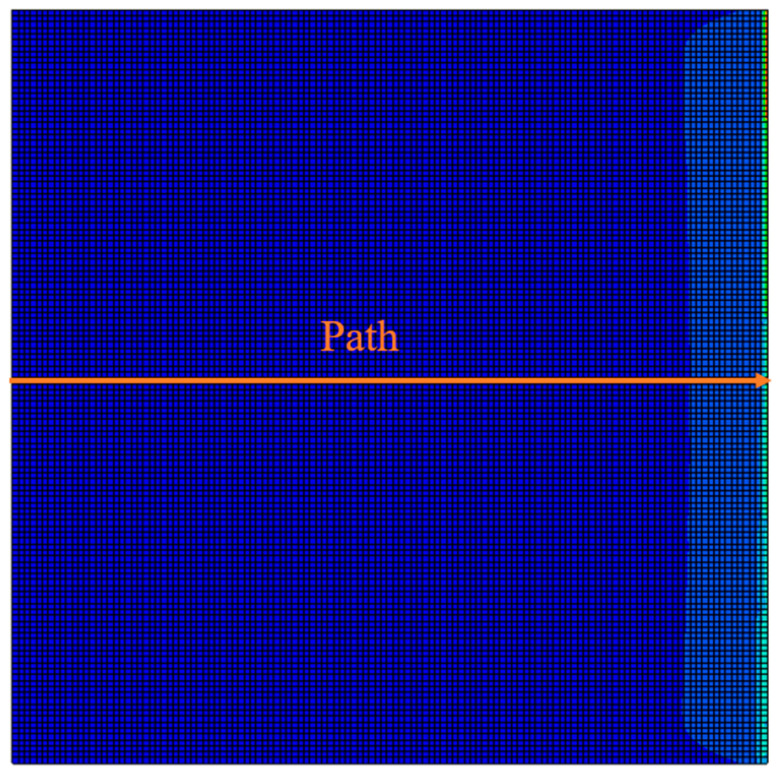
Selected nodal path for transient thermal analysis verification.

**Figure 13:**
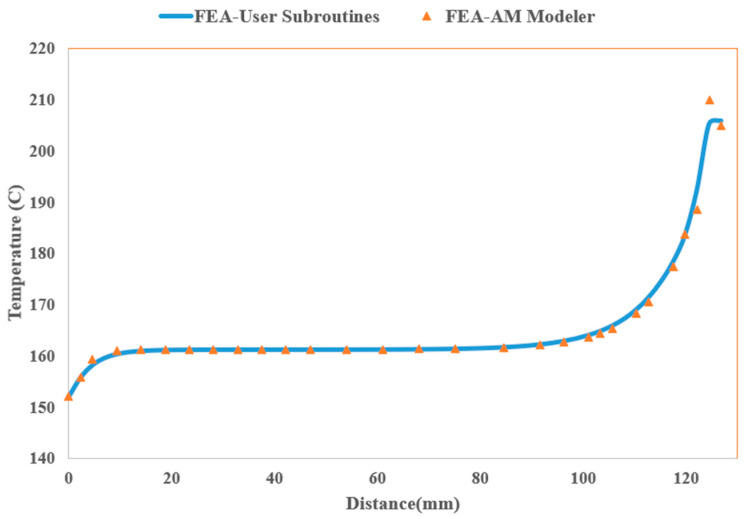
Nodal temperature distribution along the selected path.

The nodal temperature distribution at the end of the printing step, along a path illustrated in Figure 12, crossing the central portion of the top surface of the plate, is compared between the FEA-AM Modeler and FEA-User Subroutines, and the results are shown in Figure 13. Once again, a good agreement is evident in the results obtained from both procedures. As previously described, the top layer is printed starting from the origin and proceeds in the y-direction (90°) from left to right. Consequently, the nodal temperatures exhibit a gradient, with lower temperatures prevailing on the left-hand side, gradually increasing as we advance towards the right, where new material is recently deposited. The notably lower temperature near the far-left edge can be attributed to the dissipation of heat from both the top and left surfaces of the plate in this vicinity. For the middle nodes situated far from the left edge, heat loss is predominantly from the top surface, resulting in a slightly higher temperature at these nodes.

Convincing agreement between results obtained from transient thermal analysis of sample geometry from two different approaches shows accuracy and reliability of proposed method. In the following section, we discuss the application of the proposed method in the second part of our problem which is mechanical analysis.

### 4.2. Mechanical Analysis

Due to the high-temperature gradients and rapid cooling rates in the FDM process discussed in Section 4.1, polymeric materials shrink as they cool down from a molten state to ambient temperature. This shrinkage induces residual stress buildup within FDM parts that could lead to fracture and failure. In the FDM process, the impact of thermal residual stress is particularly severe due to the layered and bead-wise nature of FDM printed parts. Hence, in this section, we first validate our structural model by comparing it with existing experimental data and then focus on residual stresses and distortion in FDM printed parts.

#### 4.2.1. FE Simulation Model

In this analysis, a 120 × 120 × 5.2 mm^3^ rectangular plate made of 50 wt.% CF/PPS is considered to assess the accuracy of the proposed model. The selection of this specific geometry and material is based on the availability of associated experimental data in previous research, facilitating the validation of the proposed model. The boundary conditions and print parameters used are the same as those specified in Table 1 of Section 4.1.1. The plate consists of 4 layers, each with a thickness of 1.3 mm, printed with a layup of [0, 0, 90, 90]. The first two layers are printed in the x-direction, and the last two layers are printed in the y-direction. For mechanical analysis, the bottom nodes of the printing bed are constrained in lateral degrees of freedom in the printing and cooling steps. In the last step, which involves the cooling step off the printing bed, this constraint is removed, with only a few nodes in the middle of the plate fixed to restrict rigid body movement. 

#### 4.2.2. Verification of FE Simulation

**Figure 14:**
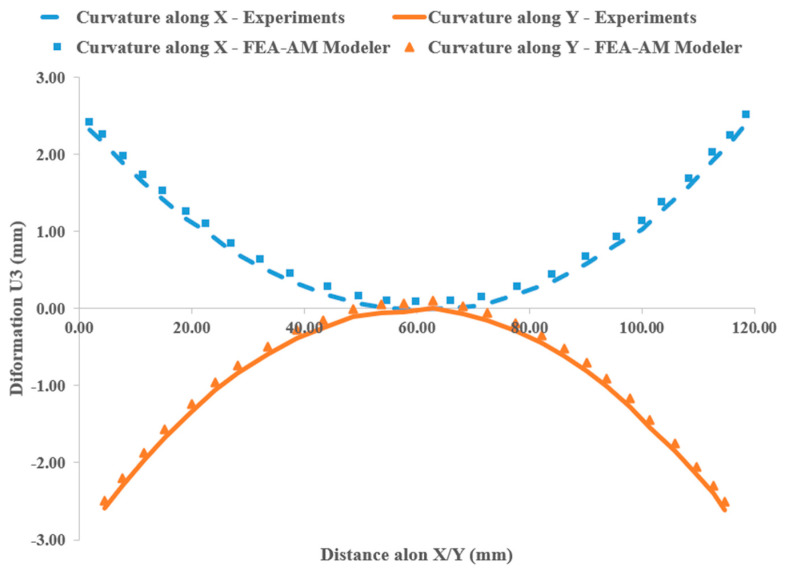
Verification of the model using available data in the literature [38]. Nodal displacement in stacking direction along the selected paths.

To validate the applicability of the proposed FEA-AM Modeler in mechanical analysis, nodal paths containing the middle nodes of the plate’s bottom face in the x-direction and y-direction are selected, and the nodal displacement in the stacking direction (U3) is measured. The results are compared with experimental data from the literature [38], as shown in Figure 14. The maximum error observed in the comparison results is less than 4%, affirming the accuracy and reliability of the proposed model. While a few nodes are laterally fixed in in the middle of the plate to account for rigid body motion, resulting in zero displacement along the selected path, two edges of the plate perpendicular to the *x*-axis are folded upward, shown in blue color with maximum deflection of about 2.4 mm, while the other two edges perpendicular to the *y*-axis are folded downward (Orange color) with maximum deflection of about 2.6 mm, previewing its deformation into a saddle shape, as discussed in the next section. 

#### 4.2.3. Residual Stresses and Distortion

In FDM printed parts, failure usually arises from thermal residual stress buildup. The 3D printed 50 wt.% CF/PPS is considered an orthotropic material with distinct thermal and mechanical properties in different directions. These directions include the printing direction (also the fiber orientation direction), the transverse in-plane direction (perpendicular to the printing direction), and the stacking (build) direction. Because of weak bonding between neighboring beads of material and enhanced properties of carbon-reinforced materials in the print direction, the stress component in the transverse in-plane direction is particularly critical and contributes significantly to failure. Therefore, particular attention is given to this stress component.

**Figure 15:**
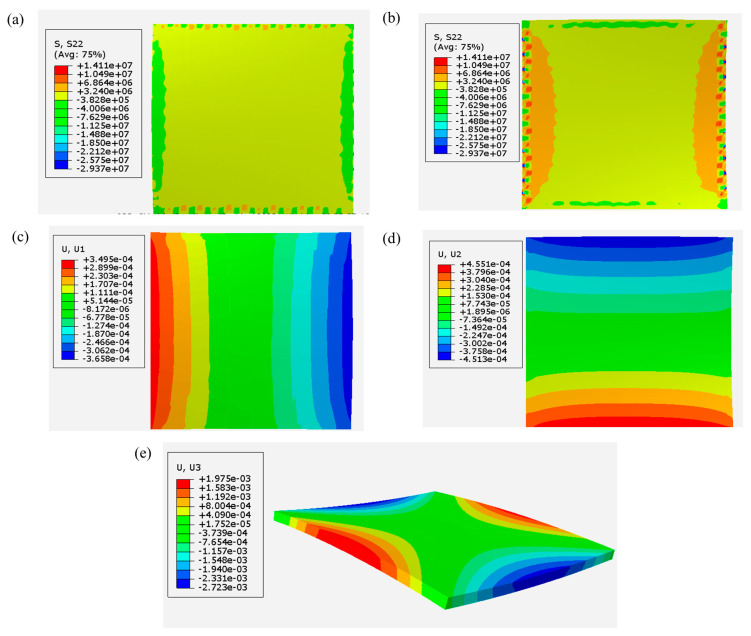
Residual stress and subsequent warpage of printed plate: (**a**,**b**) Stress components in the transverse in-plane direction on the top and bottom of the plate, respectively; (**c**) Nodal displacement in the printing direction; (**d**) Nodal displacement in the transverse in-plane direction; (**e**) Nodal displacement in the stacking direction (z).

Figure 15 shows the buildup of residual stress and the resulting warpage of the plate at the end of the cooling step-off the printing bed. Specifically, Figure 15a plots the transverse in-plane component of residual stress over the top surface of the printed plate, while Figure 15b shows the same components of stress on the bottom surface.

The printing configuration, specified as the setting of the print [0, 0, 90, 90], indicates that the first two layers are printed in the x-direction. As the stiffness of printed material is maximum in the print direction due to fiber orientation, material shrinkage is minimized in the print direction and maximized in the transverse in-plane direction. This behavior is evident in the shrinkage of the bottom face of the plate in the y-direction (transverse in-plane for the first two layers), as shown in Figure 15d. On the other hand, Figure 15c, depicting the top surface of the plate where the material is printed in the y-direction, shows that the maximum shrinkage tends to occur in the transverse in-plane direction, which is the x-direction for the last two layers of the printed plate.

Based on this analysis, it can be concluded that the top surface of the printed plate tends to shrink in the x-direction and around the y-direction, while the bottom face of the plate tends to shrink in the y-direction and around the x-direction. This shrinkage behavior results in the final saddle shape, as shown in Figure 15e. This aligned with the compressive stress observed on the top surface (S22) and the tensile stress on the bottom face of the plate, as demonstrated in Figure 15a,b, respectively. 

## 5. Conclusions

In this study, we presented a comprehensive investigation into the Finite Element Method (FEM) simulation of Fused Deposition Modeling (FDM) additive manufacturing, focusing specifically on the thermal and mechanical behaviors of semi-crystalline composite materials. Leveraging the capabilities of Abaqus software, we explored two distinct approaches: one utilizing traditional user subroutines (FEA-User Subroutines) and another integrating the Abaqus AM Modeler plug-in with selected user subroutines (FEA-AM Modeler). Through a series of rigorous validations against experimental data and existing literature, we demonstrated the accuracy and efficacy of our simulation methodologies. 

Our thermal analysis examined the intricate temperature distribution dynamics during the FDM printing process, which is crucial for understanding material behavior and shrinkage phenomena. By simulating the printing and cooling steps, we accurately captured the thermal gradients and cooling rates, validating our results against experimental temperature measurements. The agreement between simulated and experimental temperature distributions underscored the fidelity of our approach in modeling the complex thermal behavior of FDM-printed components.

Moving beyond thermal analysis, we investigated mechanical simulations to assess residual stresses and deformation in FDM-printed parts, particularly emphasizing the unique characteristics of semi-crystalline materials. Utilizing orthotropic material models and advanced constitutive equations, we accurately predicted mechanical responses and validated them against experimental displacement data. Our findings highlighted the significance of thermal residual stresses in governing part integrity, particularly emphasizing the role of material orientation and layer-wise printing configurations in inducing stress gradients and deformations.

Crucially, our study showcased the potential of the FEA-AM Modeler approach, offering a streamlined and efficient methodology for simulating FDM printing processes with semi-crystalline composite materials. By seamlessly integrating user subroutines with the AM Modeler plug-in, we demonstrated enhanced computational efficiency and ease of analysis setup without compromising accuracy or reliability. This hybrid approach represents a promising avenue for future research and industrial applications, enabling engineers to efficiently optimize FDM printing parameters and predict part performance with confidence.

Our study contributes to advancing the state-of-the-art in Finite Element simulation of FDM additive manufacturing, providing valuable insights into the thermal and mechanical behavior of printed components. By validating our methodologies against experimental data, we have established robust simulation frameworks capable of accurately predicting temperature distributions, residual stresses, and deformation in FDM-printed parts. These findings pave the way for informed design optimization and quality assurance in FDM additive manufacturing, fostering innovation and efficiency across diverse industries, particularly in the realm of semi-crystalline composite materials. While our investigations focused on two specific polymers, it is important to note that our methodology can be extended to other polymeric materials suitable for FDM printing.

## Figures and Tables

**Table 1:** Print parameters of the simulation.

Parameter	Value
Print speed	20 mm/s
Ambient temperature	25 °C
emissivity	0.97
Bed temperature	200 °C
Number of elements	11,664
Convection film coefficient	30 W/(mK)

**Table 2:** Print parameters for experiment.

Parameter	Value
Print speed	20 mm/s
Layer height	0.5 mm
Extrusion temperature	210 °C
Bed temperature	60 °C

## Data Availability

Data are contained within the article.
